# Inhibiting Endocannabinoid Hydrolysis as Emerging Analgesic Strategy Targeting a Spectrum of Ion Channels Implicated in Migraine Pain

**DOI:** 10.3390/ijms23084407

**Published:** 2022-04-15

**Authors:** Adriana Della Pietra, Juha Savinainen, Rashid Giniatullin

**Affiliations:** 1 A. I. Virtanen Institute for Molecular Sciences, University of Eastern Finland, 70211 Kuopio, Finland; adriana.della.pietra@uef.fi; 2 Institute of Biomedicine, University of Eastern Finland, 70211 Kuopio, Finland

**Keywords:** migraine, endocannabinoid, MAGL, FAAH, inhibition, nociceptors

## Abstract

Migraine is a disabling neurovascular disorder characterized by severe pain with still limited efficient treatments. Endocannabinoids, the endogenous painkillers, emerged, alternative to plant cannabis, as promising analgesics against migraine pain. In this thematic review, we discuss how inhibition of the main endocannabinoid-degrading enzymes, monoacylglycerol lipase (MAGL) and fatty acid amide hydrolase (FAAH), could raise the level of endocannabinoids (endoCBs) such as 2-AG and anandamide in order to alleviate migraine pain. We describe here: (i) migraine pain signaling pathways, which could serve as specific targets for antinociception; (ii) a divergent distribution of MAGL and FAAH activities in the key regions of the PNS and CNS implicated in migraine pain signaling; (iii) a complexity of anti-nociceptive effects of endoCBs mediated by cannabinoid receptors and through a direct modulation of ion channels in nociceptive neurons; and (iv) the spectrum of emerging potent MAGL and FAAH inhibitors which efficiently increase endoCBs levels. The specific distribution and homeostasis of endoCBs in the main regions of the nociceptive system and their generation ‘on demand’, along with recent availability of MAGL and FAAH inhibitors suggest new perspectives for endoCBs-mediated analgesia in migraine pain.

## 1. Introduction: Migraine Pain Signaling Pathways as Target for Antinociception

**Figure 1:**
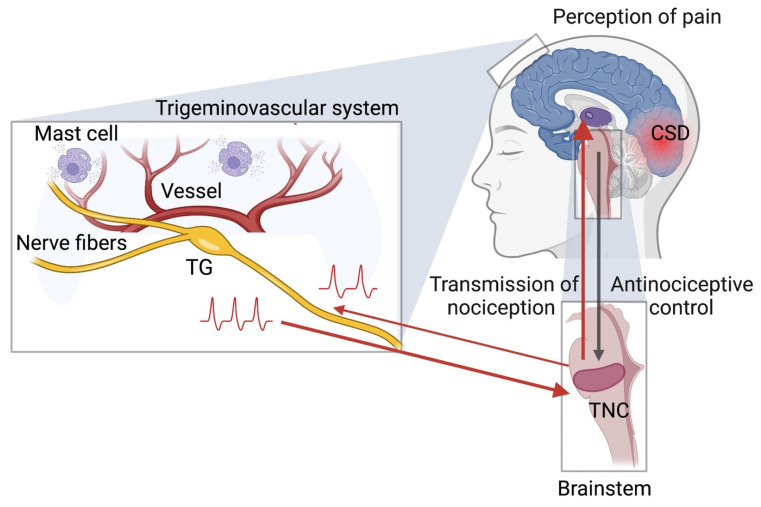
Migraine pain origin, transmission and perception. Migraine related nociceptive signalling originates in the trigeminovascular system (TGVS) composed of the trigeminal ganglia (TG), nociceptive Aδ- and C-fibres projecting to meninges, dural mast cells and the local vasculature. These structures can interact with each other via chemical or mechanical communications forming a *vicious circle*, which promotes and supports neuroinflammation, activation and sensitization of nociceptors. Nociceptive signalling (red arrows) can be initiated by mechanical forces, from pulsating dural vessels, CSD- or stress-induced degranulation of mast cells or by antidromic spiking directed to the meninges and associated with the release of several neuropeptides including CGRP. Migraine-related nociceptive signalling is transmitted from the meninges through the brainstem (zoomed down in grey box) trigeminal nucleus caudalis (TNC) and thalamus (purple), to the higher pain centres in the cortex performing the function of pain perception. Opposite to the ascending nociceptive signalling, the descending inhibitory control of the brainstem provides the anti-nociceptive function (dark grey arrow). A migraine attack can start with cortical spreading depression (CSD), a phenomenon typical for migraine with aura with massive depolarization of neurons and glial cells slowly propagating along the cortex.

Migraine is a primary headache disorder in which one of the worst symptoms is the severe throbbing pain [1]. The molecular mechanisms underlying migraine pain are still mostly unknown, but current evidence supports the involvement of both central and peripheral mechanisms in this common neurological disorder [2,3]. It is widely accepted that migraine pain originates from the meninges in the trigeminovascular complex composed by nociceptive Aδ- and C-fibers, projecting from the trigeminal ganglion (TG) and innervating local vasculature and connective tissues in the meninges (Figure 1) [4]. This local trigeminal nerve terminals can release the neuropeptide calcitonin gene-related peptide (CGRP), which plays a central role in migraine pain and represents the important target for anti-migraine interventions [5]. In addition, there is a release of histamine, serotonin and cytokines from mast cells, ATP and nitric oxide from endothelial cells, substance P and acetylcholine from the peripheral nerve terminals fibers [6,7,8,9,10]. These pro-nociceptive events can be initiated by different triggers. Among them are mechanical forces coming from pulsating intracranial vessels, which can activate mechanosensitive Piezo1/2 receptors expressed in the meningeal afferents and degranulation of multiple meningeal mast cells, which can be initiated by stress or by cortical spreading depression (CSD) [11,12,13,14,15]. Moreover, the release of CGRP and the degranulation of mast cells could be induced by antidromic spiking, which comes from central to peripheral nerve endings in the meninges [12]. Most of these local pro-inflammatory molecules can directly activate and sensitize meningeal peripheral nerve endings, making them highly susceptible to chemical and mechanical stimuli [13].

Together, these pro-inflammatory and pro-nociceptive molecules released by interacting nerve fibers, vessels and immune cells are forming a sort of *vicious circle*, which further promotes the sustained state of inflammation, persistent activation and sensitization of nociceptors [14]. Blocking the release of CGRP represents one of several possible mechanisms to disrupt this pro-nociceptive vicious circle. Likewise, this positive pro-nociceptive loop can be broken by the stabilization of local mast cells, which form a neuro-immune synapse with trigeminal nerve endings [10].

Apart from the important role in the initiation of migraine pain in peripheral meningeal afferents, there are studies proposing a pro-nociceptive role of somas of trigeminal neurons located in the ganglion and cross-talking with the surrounding satellite glial cells [16,17]. Interestingly, the release of CGRP from meningeal fibers and from somas of neurons in the trigeminal ganglion can be differently sensitive to the inhibitory action of anti-migraine drugs, such as the agonists of serotonin 5-HT1 receptor [18]. Together, these data suggest that, at the periphery, there are two distinct triggering zones for migraine pain (Figure 1).

However, despite the fundamental role of the peripheral structures, long-lasting headache also involves the central mechanisms, which, finally, results in central sensitization [2,3]. Such a broad view can better explain the whole spectrum of phenomena typical for migraine, which in many senses is similar to other diffused chronic pain conditions [19]. In the CNS, the brainstem trigeminal nucleus caudalis (TNC) collects and further transmits the incoming nociceptive signals from meninges to the thalamus (Figure 1) and then, to the anterior cingulate cortex (ACC), amygdala and insular cortex, the structures related to the emotional perception of migraine pain [20]. On the other hand, the descending anti-nociceptive control of the brainstem can counterbalance and eventually block the nociceptive traffic from the periphery (Figure 1) to keep the ‘gates’ for pain signaling closed in normal conditions but, probably, open them during the migraine attack [10].

The early involvement of cortical areas in migraine pathology takes place in the less frequent form of migraine with aura, which typically starts with the development of CSD (Figure 1), a wave of strong depolarization of cortical neurons and glial cells [21]. This is an example of one of the key migraine events when the origin of the attack is localized within the CNS. Brain oedema, associated with CSD [21], can mechanically compress the meningeal tissues, facilitating the activation of mechanosensitive Piezo1/2 channels in local nerve fibers [11]. From the therapeutical perspective, CSD represents a therapeutic target for damping down the harmful hyperexcitable neuronal state, associated with elevated glutamate release [22,23].

To summarize, migraine pain is initiated and supported by interactions between the peripheral meningeal nociceptive system, brainstem network and central pain centers [24]. Thus, migraine pain can potentially be blocked at different levels by targeting distinct structures and receptor systems specifically expressed within these structures. A deeper knowledge of location and the leading mechanism of the multicomponent migraine pain may give a chance to block pain most efficiently in a personalized manner in a given migraine patient.

Figure 1 illustrates pain triggering peripheral zones and several relay stations for pain generation and transmission, which is finally culminating in the CNS. For the heterogeneous in nature migraine, acute and prophylactic pharmacotherapy [3,25] may work differently in distinct patients according to the prevailing involvement of distinct pain-related structures. The most clear example, which requires a specific approach, is migraine with aura, where the main aim of preventing therapy is the reduction of cortical hyperexcitability. Currently, the field of personalized medicine is under active development and effective treatments such as new types of 5-HT1 agonists, CGRP receptor inhibitors, recently approved anti-CGRP monoclonal antibodies and botulinum neurotoxin serotype A (reviewed in [5,26]) suggest a spectrum of various promising therapeutic strategies. However, despite clear progress with these innovative approaches, many patients still remain untreated [26], demonstrating a need for more innovative types of migraine therapy.

Apart from the synthetic antimigraine drugs mentioned above, an alternative strategy could be to enhance the efficiency of endogenous protective mechanisms inhibiting pain. For this aim, the natural anti-nociceptive drive mediated by serotonergic and noradrenergic agents, endogenous opioid system, or other endogenous molecules and inhibitory neuronal networks can be employed [10]. Relying on this strategy, in this review, we aimed to show promising perspectives of engaging the endogenous endocannabinoid system (ECS) in order to inhibit migraine pain at its origin sites or key points of transmission of nociceptive signals to the higher pain centers.

## 2. ECS in Anatomical Structures Important for Migraine Pain Signaling

### 2.1. Main Components of the ECS as a Target for Analgesia

**Figure 2:**
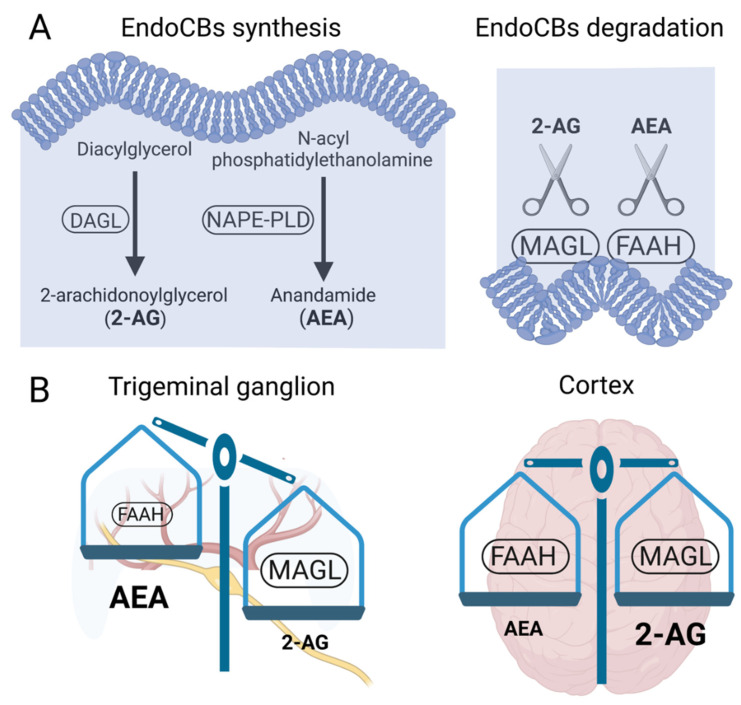
EndoCBs synthesis, degradation and distinct MAGL and FAAH profiles in migraine pain pathways. (**A**) Main enzymatic steps of 2-AG and AEA synthesis and degradation. (**B**) Text size of enzymes and endoCBs is used to emphasize the relative enzymatic activity and endoCBs levels in the trigeminal ganglion and in cortex. On the left, MAGL, in contrast to FAAH, is the prevalent endoCBs hydrolysing enzyme in the trigeminal ganglion, whereas, in the brain (on the right), both MAGL and FAAH are highly active. Despite the high active state of both FAAH and MAGL in the brain, due to higher synthesis, the basic level of 2-AG in the brain is much higher than that of AEA. In contrast, in the trigeminal ganglion, the level of AEA appears to be high due to lower FAAH activity.

In general, the ECS works as a homeostatic regulator in essentially all organ systems to control many physiological processes, including nociception [27]. ECS is composed by the primary endoCBs 2-arachidonoyl glycerol (2-AG) and *N*-arachidonoyl ethanolamide (alias anandamide, AEA) and their synthetic enzymes diacylglycerol lipase (DAGL) and NAPE-specific phospholipase D (NAPE-PLD), respectively. There are also endoCBs degrading enzymes monoacylglycerol lipase (MAGL) and fatty acid amide hydrolase (FAAH) and at least two G-protein-coupled CB1 and CB2 receptors, mediating the signaling induced by endoCBs [28]. Figure 2A shows the main steps in the synthesis and degradation of endoCBs. The primary endoCB 2-AG is produced locally, on demand, according to the intensity of the neuronal activity, from the membrane lipid precursors as a result of activation of phospholipase C (PLC) in cells that also express DAGL [29,30,31]. DAGL converts the PLC product diacylglycerol (DAG) into 2-AG or another monoacylglycerol, called 2-oleoylglycerol (2-OG) [29]. 2-AG is degraded by enzymatic hydrolysis into glycerol and free arachidonic acid by several enzymes, primarily, by the membrane attached presynaptic MAGL (Figure 2A), but also by the recently identified alpha-beta hydrolase domain proteins (ABHD6, and ABHD12) [29,32,33]. Instead, AEA and other *N*-acyl ethanolamines (NAEs), such as palmitoylethanolamide (PEA) and oleoylethanolamide (OEA), are synthesized from *N*-acyl-phosphatidylethanolamine (NAPE) by NAPE-PLD, [29,34]. AEA, like other NAEs are hydrolyzed by FAAH, which is also a membrane-bound enzyme (Figure 2A) [29], and *N*-acylethanolamine-hydrolyzing acid amidase (NAAA), which is typically more active in peripheral tissues [35].

Apart from the enzymatic degradation, extracellular endoCBs levels are maintained physiologically low presumably by uptake processes whose nature remains not fully resolved [29]. Indeed, AEA sequestration has been associated with different mechanisms mediated by fatty acid binding proteins (FABPs) [36], heat shock proteins [37], sterol carrier protein 2 [38] located in lipid rafts [39], or bidirectional membrane transporters [40]. It is under investigation whether similar mechanisms also regulate 2-AG uptake and/or sequestration [41].

The ECS is involved in performing several vital functions in both the CNS and periphery, including the modulation of excitability and neurotransmission via presynaptic CB1 receptors and the regulation of the immune system, mainly through CB2 receptors. Recently, the ECS has been considered as one of the main targets for achieving analgesia in chronic pain [42]. This type of analgesia could be a desirable alternative to opioids, which produce an effective pain relief but at the expense of several serious side effects, including psychotropicity, tolerance and addiction [43]. Thus, a range of cannabis-related chemical tools have emerged recently, including phytocannabinoids, synthetic cannabinoids and endoCBs [44]. Among them, endoCBs are especially attractive as they are naturally produced locally and ‘on-demand’ in the key regions of the nociceptive system and, due to ther physiological properties, have less side effects than plant cannabinoids. Some studies have already revealed that the enhanced levels of 2-AG and AEA in certain areas of the nervous system after inhibition of their respective degrading enzymes, MAGL and FAAH, produced analgesic effects almost free of side effects [45].

More detailed description of MAGL- and FAAH-targeted analgesia via endoCBs is presented in the Section 3 and Section 4.

### 2.2. MAGL and FAAH Activity in Migraine-Related Areas of the Nervous System

The endoCBs-degrading enzymes MAGL and FAAH are expressed in structures related to pain origin, nociceptive transmission and perception of pain (Figure 2B) [46,47]. However, the relative activity of these two enzymes, the major factor determining the functional role of 2-AG and AEA as endogenous analgesics, is not equally present in the PNS and CNS. As shown in Figure 2B, endoCBs hydrolysis, MAGL and FAAH, are differentially active in the trigeminal ganglion, which is a part of the peripheral nociceptive system and in the brain areas, where pain is finally perceived [47]. Indeed, based on the activity-based protein profiling method (ABPP), identifying active serine hydrolases, including MAGL and FAAH, we found that, in the trigeminal ganglion, the MAGL activity is much higher than that of FAAH (Figure 2B) [47]. Likewise, the level of endoCBs at the periphery is expected to be non-equally present in favor of accumulated AEA, while the amount of 2-AG should be basically low due to the active degradation by MAGL. Notably, this imbalance could be changed by the inhibition of MAGL activity. Thus, in the trigeminal ganglion, the MAGL/2-AG axis is a highly tunable target for pharmacological interventions aiming to reduce peripheral mechanisms of migraine pain through enhanced level of endoCBs.

In contrast to the peripheral trigeminal nociceptive system, FAAH and MAGL activity is comparable at the cortical level (Figure 2B) [47]. Thus, in the CNS, the dual inhibition of these two endoCBs degrading enzymes could be an attractive option in order to reduce the central transmission of migraine-related pain signalling. There is, however, clear evidence that, in the CNS, the level of 2-AG is much higher than AEA [48], suggesting the leading role of 2-AG in the ‘natural’ modulation of pain processing in the brain. Indeed, the high 2-AG synthesis can be achieved in the brain after increased neuronal activity by following enhancement of phospholipase C (PLC) and diacylglycerol lipase (DAGL) activities along with the rise of calcium in neurons and in astroglia, making the synthesis of 2-AG greater than the AEA one [49]. Notably, even the similar level of endoCBs at the same location does not predict their equal activity, as, for instance, AEA is a partial agonist at CB1/CB2 receptor, while 2-AG is a full agonist at both receptor types [50].

The inhibition of MAGL, the main 2-AG degrading enzyme at the periphery (Figure 2) [47], represents a potential mechanism for blocking the early events in the transmission of migraine pain. However, the sustained nociceptive signalling in the meningeal trigeminovascular system could be modulated by AEA acting on local immune cells [51]. Thus, dura mater is enriched with mast cells [6,52], where their degranulation can trigger a nociceptive cascade of signalling in trigeminal afferents via the release of serotonin [8,10,53]. Notably, one of the analogs of AEA, methanandamide, inhibits the degranulation of dural mast cells through CB2 receptors [53], supporting the notion that these immune cells might also be a target for raised endoCBs, in particular, to AEA. Therefore, various treatments promoting 2-AG and AEA signalling at the local environment, surrounding meningeal afferents, can potentially reduce the generation and transmission of pain to the second order brainstem neurons [54]. In conclusion, in addition to the evident role of 2-AG, there are data showing the role of FAAH/AEA-mediated signaling as a target for peripheral analgesia.

To summarize, endoCBs with their specific receptors, synthesizing and degrading enzymes are widely but not equally expressed in structures involved in migraine pain generation, transmission and perception [47,55,56]. Thus, the selective enhancement of 2-AG and AEA via MAGL and FAAH inhibition, respectively, can provide a beneficial reduction of pain triggering, transmission and excessive cortical excitability, underlying migraine pathophysiology.

## 3. EndoCBs Control of Nociception via Cannabinoid Receptors and through the Direct Action on Ion Channels

### 3.1. Distribution of CB1 and CB2 Receptors and Retrograde endoCB Signaling in the Nociceptive System

According to the traditional view, endoCBs mediate their physiological effects via two main inhibitory Gi/o-protein-coupled cannabinoid CB1 and CB2 receptors [28]. Both in the CNS and the periphery, the modulation of neurotransmission is mainly mediated by neuronal presynaptic CB1 receptors [54]. CB1 receptors are specifically abundant at the central neuronal networks [57]. In contrast to CB1, CB2 receptors are widely presented in the immune cells, enriched in the meninges, as well as in microglia, but they are also found in brainstem neurons [45,58,59]. It is important that, unlike adenosine, which selectively blocks the release of glutamate but not of GABA [60], the activation of CB1 receptors inhibits transmitter release from both GABAergic and glutamatergic neurons [61,62,63,64].

**Figure 3:**
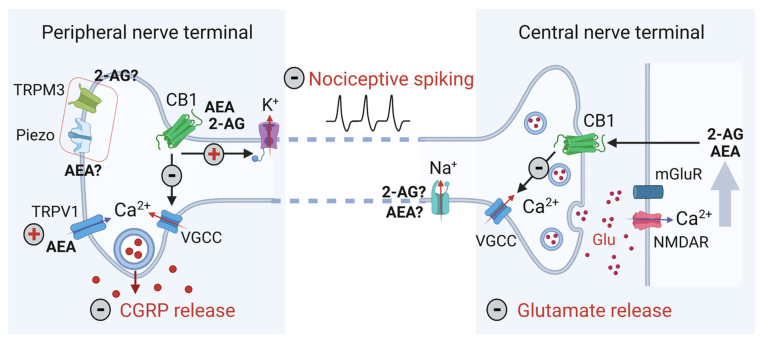
EndoCBs interfere with multiple ion channels in peripheral migraine pain mechanisms. Nociceptive spiking, Ca^2+^-dependent CGRP release in the peripheral nerve terminal (**left**), and glutamate release in the central nerve terminal (**right**), are the main targets for endoCBs leading to pain inhibition. In the peripheral nerve terminal, the activation of CB1 receptors by endoCBs results in the inhibition of voltage gated calcium ion channels (VGCC), resulting in reduced CGRP release. The CB1-mediated opening of potassium ion channels reduces excitability and diminishes nociceptive spiking. AEA also acts as a direct agonist of TRPV1 receptors, thus opposing peripheral anti-nociception via CB1 mechanism. Peripheral terminals also express mechanosensitive TRPM3 and Piezo ion channels (in the red box), which can potentially be modulated by endoCBs through modifications of the lipidic environment. In the central nerve terminal, glutamate release stimulates endoCBs synthesis by postsynaptic Ca^2+^ influx through NMDA receptor and PLC enhancement following mGluR activation. EndoCBs retrogradely approaching presynaptic terminals reduce glutamate release by blocking VGCC. The action of endoCBs is mediated by CB1 receptors but they can also work as allosteric modulators, directly targeting sodium ion channels and thus, further affecting the generation and propagation of nociceptive spikes. Plus (+) and minus (−) symbols indicate the enhancement or inhibition of ion channels by endoCBs, respectively.

Figure 3 shows that, in the primary nociceptive afferents, activation of CB1 by endoCBs results in the inhibition of CGRP release from peripheral terminals, while in the central processes, endoCBs are blocking glutamate release, which mediates transmission of nociceptive signals to the second order neurons in the TNC [65]. Thus, a combination of these two inhibitory effects of secretion provides an added value for the anti-nociception by endoCBs.

Within the CNS, endoCBs are produced locally at the postsynaptic membranes from where they are released and trans-synaptically travel, in a retrograde manner, to activate presynaptic CB1 receptors. Indeed, depolarization-induced suppression of transmitter release in excitatory and inhibitory synapses, called DSE/DSI, mediated by retrograde endoCBs signaling, is a well-studied phenomenon in the CNS [64,66,67]. Notably, in the phenomenon of DSE, the role for 2-AG is much more important than the one of AEA [68], consistent with its leading role in the control of synaptic transmission.

The anti-nociceptive potential of cannabinoid CB1 receptors is well established [69,70]. In the synapse coupling the primary afferent with the second order nociceptive neuron (Figure 3), glutamate, via metabotropic mGluR receptors, enhances the activity of phospholipase C (PLC), which, in turn, stimulates 2-AG synthesis by DAGL from the precursor molecule diacylglycerol (DAG) [71]. Calcium influx, promoted mainly by post-synaptic NMDA receptors, further supports 2-AG and AEA synthesis from the membrane lipid precursors [72]. Together, these concerted actions represent an efficient endogenous negative feedback mechanism limiting pain signal transmission in a use-dependent manner. Notably, the performance of this mechanism of autoinhibition also critically depends on the activity of MAGL and FAAH, which limits the level of both endoCBs. In addition to the signaling via neuronal CB1 receptors, at the spinal and supraspinal parts of the CNS, endoCBs can suppress pain by acting via glial CB2 receptors [54].

At the molecular level (Figure 3), activation of presynaptic CB1 receptors, operating via inhibitory Gi/o-proteins, by blocking presynaptic voltage-gated calcium-channels, inhibits release of glutamate as well as CGRP, from the presynaptic neuron [73]. Moreover, the activation of CB1 receptors has been linked to the opening of inward rectification potassium channels [74]. These channels contribute to the maintenance of the resting membrane potential and their activation should reduce the neuronal excitability as an additional anti-nociceptive mechanism (Figure 3). CB1 receptors’ activation also leads to decreased cAMP levels and to PKA inhibition [75], thus reducing the neuronal sensitization. Together, these numerous complementary mechanisms determine a multicomponent anti-nociceptive effect of endoCBs.

### 3.2. Pro-Nociceptive Effects of EndoCBs via TRPV1 Receptors

In addition to interaction with the canonic inhibitory CB1 and CB2 receptors, endoCBs are able to engage the noncannabinoid receptor-mediated neuromodulation. For instance, AEA has been reported to activate, although at high concentrations, the transient receptor potential vanilloid receptor (TRPV1), which may trigger CGRP release and promote nociceptive signaling (Figure 3) [76,77]. Thus, the TRPV1 receptor, which is forming a calcium-permeable ion channel, can function as an ionotropic cannabinoid receptor under both physiological and pathological conditions [50]. In the context of migraine, TRPV1 receptors are highly expressed in nociceptive meningeal afferents [78]. These receptors are also detected in other migraine related areas such as the spinal cord, thalamus, cerebellum, cortex, and limbic system [79,80]. Notably, while the action of AEA via CB1 receptors represents an antinociceptive effect due to the reduced release of glutamate as well as of substance P and CGRP (Figure 3) [81], the final functional outcome of interactions between AEA and TRPV1 receptors in in vivo conditions remains unclear. Interestingly, endoCBs-mediated CB1 activation can decrease the sensitivity of TRPV1 receptors [46], thus potentially reducing pain [82]. Nevertheless, as higher AEA concentrations can be achieved locally after a complete inhibition of FAAH, the resulting AEA interaction with TRPV1 receptors should be taken into consideration when planning treatment options based on raised levels of both endoCBs.

### 3.3. Modulation of Nociception by EndoCBs via Membrane Lipid Environment and Direct Interaction with Ion Channels

Meningeal afferents in the trigeminovascular system express many pain-related ion channels. In addition to the well-established interaction of AEA with TRPV1 receptors, there are potentially more molecular targets for AEA and 2-AG among the plethora of ion channels shaping nociceptive signaling in meningeal C- and Aδ fibers. Thus, nociceptive spike generation and propagation primarily depends from sodium ion channels, which profile is specific for C- and Aδ fibers [83,84]. Nociceptors also widely express ATP-gated P2X receptors [85] and recently discovered mechanosensitive Piezo1/2 channels [11,86,87] as well as sex hormones sensitive TRPM3 receptors [88].

The activity of most of these transmembrane channels, primarily of mechanosensitive gigantic Piezo proteins, largely depends on the profile of membrane lipids, in particular, on the level of phosphatidylinositol 4,5-bisphosphate (PIP2) [89] and specific fatty acids [90]. Mechanosensitive channels are of special interest in the context of migraine, as this disorder is associated with such symptoms as allodynia, mechanical hyperalgesia and pulsating pain [11,86]. Given the lipid nature of endoCBs and their link to the lipid profile of the membrane, in particular, their transformation to arachidonic acid (AA), it is likely that ECS activity can modulate mechanosensitive ion channels through this noncanonical signaling. If proven, such modulation of mechanosensitive TRPM3 and Piezo receptors by endoCBs via membrane lipids, analogous to the AA-mediated control of mechanosensitive K2P channels [91], could be a novel mechanism of neuromodulation which deserves further exploration.

Apart from the lipid environment of the ion channels, endoCBs potentially can serve as allosteric modulators, directly targeting ion channels to deliver the diverse functional effects [27,92]. Of key importance for the generation and propagation of nociceptive spikes is the ability of endoCBs to affect certain subtypes of potassium and sodium channels, either via CB1 receptors., or independently from CB1 activity (Figure 3).

In line with this anti-nociceptive mechanism, cannabidiol (CBD), one of the key phytocannabinoids, acts as an inhibitor of Na_V_ channels [93]. Whether endoCBs mediate a similar direct effect in order to dampen the nociceptive action potentials in trigeminal afferents is poorly explored. However, it has been found that AEA can prevent the activity of Na_V_ and L-type calcium channels in rat ventricular myocytes [94]. Consistent with the direct action on ion channels, 2-AG has been found to decrease sodium currents in frog parathyroid cells that lack CB1 and CB2 receptors [95,96].

Potassium channels are presented as a large family of membrane proteins, which have different properties directed, in general, to stabilize the membrane potential and limit or prevent spike generation. The typical coupling of CB1 receptors to opening of inward rectifying potassium channels (Figure 3) has been extended recently to show that endoCBs have mechanisms of action on potassium channels other than as cannabinoid receptors. Thus, the recent review by Lin [27] combined data demonstrating that BK, I_A_, KATP, TASK-1 and potassium channels can be the targets for cannabinoid receptor independent modulation.

In trigeminal neurons, AEA did not affect the P2 × 3 receptor, but down modulated the inhibitory GABA A receptors, which operate via the opening of chloride ion channels to prevent excitation [97]. The latter might indicate that, at the brainstem or in other parts of the CNS, accumulation of AEA might be associated with reduced GABAergic inhibition, adding more complexity in the action of endoCBs in the central synapses.

Further investigation into the molecular mechanisms underlying the direct and indirect interactions between endoCBs and ion channels is needed for improving the efficiency and selectivity of endoCB-based therapies [27].

## 4. MAGL and FAAH Inhibition to Treat Migraine Pain

### 4.1. Current Approaches to Treat Migraine Pain and the Need for New Treatment Options

In the clinical setting, modern medications directed against migraine pain can abort a migraine attack when it starts but their use is often associated with side effects and, eventually, can result in medication overuse symptoms [98,99]. Frequently administered acute migraine treatments such as triptans, ditans and opioids still have numerous side effects [10,99,100]. In most chronic migraine patients, an alternative preventive treatment is needed, including β blockers [101], anticonvulsants [102,103] and calcium channel blockers, which are effective also in targeting aura symptoms [104]. Innovative preventive strategies for management of migraine are permanently under development, both in clinical trials and in preclinical research. New, already approved options include CGRP antagonists and CGRP antibodies [105,106], as well as drugs targeting serotonin receptor subtypes [10,107]. In the meantime, ECS is already discussed as an additional approach to modulate chronic pain [55,108].

Based on recently established data on the activity of endoCBs hydrolyzing enzymes in the migraine related areas of the PNS and CNS [47], the possibility to engage ECS for the treatment of migraine pain is now getting stronger support.

### 4.2. Preventing Endocannabinoid Hydrolysis as a Novel Analgesic Strategy

The selective enhancement of AEA and 2-AG levels in the tissues can be achieved by administration of the MAGL or FAAH inhibitors, respectively. An efficient and specific MAGL and FAAH inhibition should prevent 2-AG and AEA hydrolysis, thereby increasing their levels in the nervous system and other migraine related tissues. The raised levels of endoCBs can provide a multitude of anti-nociceptive effects counteracting key events in migraine pathogenesis discussed above. An additional anti-nociceptive benefit from inhibition of AEA and 2-AG hydrolysis relies on the fact that it is diminishing the levels of their degradation product AA and its pro-nociceptive downstream products such as PGE2, as well as endovanilloids hydroxyeicosatetraenoic acid (HETE) and hydroperoxyeicosatraenoic acid (HPETE), the lipid agonists of TRPV1 receptors. It should also be noted that the activity of MAGL and FAAH could be changed by oxidative stress and during neuroinflammation [109,110], conditions which contribute to migraine pathology.

There is a continuous ongoing progress in the development of pharmacological agents which can serve as the specific FAAH or MAGL inhibitors, as well as a small group of dual inhibitors targeting both enzymes. The spectrum of recently established inhibitors in shown in Table 1. The first reported FAAH inhibitors, oleoyl and arachidonoyl derivatives of trifluoromethyl ketones and fluorophosphonates, were structurally similar to the natural substrates, giving a relatively strong but very unspecific effect due to the inhibition of several different hydrolases [111]. Later, more effective and potent FAAH inhibitors were developed, including a reversible compound OL-135 [112,113,114], irreversible URB597 [112,113,115,116] and PF3845 [112,117], which all have an analgesic effect (Table 1). In particular, the FAAH-inhibitor OL135 was efficient in a rat model of neuropathic pain, increasing AEA levels in the whole brain and in the spinal cord [114]. Its antinociceptive effect was likely based on its dual activity by targeting CB1 receptors as well as promoting desensitization of TRPV1 ion channels [118]. PF3845 also reduced pain and mechanical allodynia in the model of inflammatory pain [119,120]. The general FAAH inhibition by URB597, as well as the peripheral FAAH inhibition by URB937, reduced migraine related NTG-induced trigeminal hyperalgesia (Table 1) [121,122]. These encouraging results increased the interest in developing the FAAH inhibitors as analgesic drugs, and stimulated exploration of even more efficient and selective inhibitors. Other recently published potent FAAH inhibitors include JNJ-1661010, AKU-009, AKU-010 [123] and JZP327A [124], which have not yet been tested in migraine pathophysiology.

During the past years, the FAAH inhibitors were considered as more attractive because of their high selectivity and availability [45,112]. However, MAGL inhibitors have acquired importance because of their higher relative potency and the important role of MAGL substrate 2-AG [111]. The high expectations are also related to the elevated activity of MAGL in certain areas of the nociceptive system [47] and lead, among other endoCBs, the functional role of 2-AG signaling in the brain. Interestingly, in one of the recent reports, 2-AG was proposed to be degraded by both MAGL and FAAH [125]. However, in contrast to the inhibition of MAGL, it seems that FAAH inhibition is not able to increase 2-AG levels in the brain [126]. The latter is further supported by in vitro studies [28]. Among the first reported MAGL inhibitors was *N*-arachidonoyl maleimide (NAM), which produced an irreversible effect with low specificity [127], as well as the non-selective MAGL inhibitors methyl arachidonoyl fluorophosphonate (MAFP) and arachidonoyl trifluoromethyl ketone (Table 1) [111,127,128].

The majority of MAGL inhibitors reported thus far lack high specificity, and most of them are non-specific with regard to also affecting other hydrolases [111]. Focusing on more selective MAGL inhibitors to be used for migraine pain treatment, URB602 [129] and JZL184 [130] are able to reduce trigeminal hyperalgesia in rat NTG models of migraine (Table 1) [131]. The well-studied inhibitor JZL184 was shown to be highly specific to target MAGL, as well as KML29 [130]. They both are inducing an important analgesic and anti-allodynic effect in vivo (Table 1) [45,132,133,134]. In particular, JZL184 had a strong behavioral and peripheral antinociceptive effect on the formalin pain model [135,136] and in other neuropathies [137]. Another MAGL-inhibitor, MJN110 (more potent than JZL184 in MAGL inhibition) [138], was highly potent in attenuating mechanical allodynia and thermal hyperalgesia in neuropathic pain models (Table 1) [139]. However, MJN110 was never tested in migraine pain models.

Interestingly, FAAH can often be partially inhibited by many MAGL inhibitors [45,111]. This multiple targeting, typical for MAGL inhibitors, could represent an advantage, since it has been hypothesized that the double inhibition of MAGL and FAAH could be more effective than a complete inhibition of only one of these enzymes [140]. It was recently reported that the specific MAGL inhibitor JJKK-048 has a very high potency in vitro (IC_50_ < 0.4 nM) [141]. Therefore, it might be considered as a potential drug candidate for migraine pain treatments [47]. Given a relatively high activity in the brain (Figure 2), it appears that the FAAH inhibition has the potential to be targeted primarily in the CNS and to increase the level of AEA in order to activate neuronal CB1 receptors, which are highly expressed in the brain and spinal cord [142]. Instead, MAGL inhibitors increasing the levels of 2-AG, a full agonist of CB1 and CB2 receptors, are able to achieve its anti-nociceptive effects both in the central and peripheral nervous systems [45,58,59].

A powerful tool for targeting both MAGL and FAAH in either the trigeminovascular system or in the CNS is the recently developed dual MAGL/FAAH inhibitor AKU-005, which shows a high activity even at nanomolar concentrations (IC_50_ value 0.2–1.1 nM) [141]. Consistent with the concept of dual inhibition, the well-established dual MAGL/FAAH inhibitor JZL195 has already demonstrated its ability to relieve inflammatory pain and reduce trigeminal hyperalgesia [137,143,144,145].

Finally, it should be noted that the full inhibition of both key endoCBs degrading enzymes can potentially be associated with so-called cannabimimetic effects including catalepsy, hypothermia and hypomotility, and a desirable aim consists in the pattern of MAGL and FAAH inhibition that provides a sufficient level of analgesia without such side effects [134].

### 4.3. ECS as a Target for Treating Migraine with Aura?

Because of its specific mechanisms related to the generation of CSD, which is linked to neuronal hyperexcitability [146], migraine with aura needs the particular tools to reduce the hyperexcitable state of the cortex. The ability of cannabinoids to reduce the release of glutamate may suggest that the activation of the ECS modulates this type of migraine-related event. Although not sufficiently explored, this field of research remains controversial. Thus, one study revealed that either AEA or the CB1/2 agonist WIN 55,212-2 do not affect characteristics of CSD elicited by high potassium application [97]. The other study showed, however, that WIN55.212-2, inhibited the amplitude, duration and velocity of CSD propagation, while JWH 133, a CB2 receptor agonist, devoid of any effects in this phenomenon [147], highlights the leading role of CB1-mediated signaling in the control of neuronal mechanisms underlying CSD. The latter results suggest that CSD might be sensitive to CB1 activation, which fits with their role in reducing glutamate release from presynaptic sources, as described in the previous sections of this review. There are also studies describing functional interactions between CB1 and NMDA receptors [148], which play a key role in CSD generation and propogation [149]. Likewise, there is a report on the functional interaction between endoCBs and the activity of kynurenic acid, an endogenous NMDA receptor antagonist [150].

However, whether the recently developed endoCBs hydrolase inhibitors are also effective in counteracting CSD hyperexcitability in migraine with aura remains unexplored. Therefore, based on our recent findings of the high activity of both MAGL and FAAH in the highly excitable occipital cortex [47], there is an attractive possibility to test whether CSD could be reduced by the dual inhibition of MAGL and FAAH. If proven, this could extend the therapeutic potential of MAGL/FAAH inhibition to migraine with aura.

**Table 1:** Activity of MAGL and FAAH inhibitors tested for their analgesic effects.

Inhibitors	Compounds	IC_50_	Analgesic Effects and Targets	Ref
FAAH	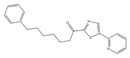 OL135	5 nM	Attenuation of mechanical and cold allodynia	[112]
	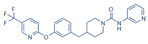 PF3845	514 nM	Attenuation of mechanical and cold allodynia	[119,120,151]
	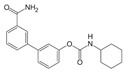 URB597	5 nM	Moderate thermal antinociception. Anti-allodynic effect in inflammatory pain. Decreased hyperalgesia in the TGVS	[115,116,122]
	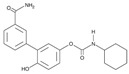 URB937	26.8 nM	Inhibition of nocifensive behavior. Decreased peripheral nociception	[121,152]
MAGL	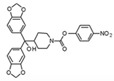 JZL184	262 nM	Behavioral analgesic effects. Reduction of NTG-induced hyperalgesia of spinal and TGVS origin	[130,131,135,136]
	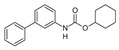 URB602	280 nM	Reduction of NTG-induced hyperalgesia of spinal and TGVS origin	[129,131]
	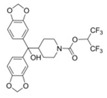 KML29	43 nm	Behavioral analgesic effect	[45,132,133,134]
	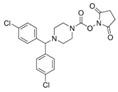 MJN110	<100 nM	Attenuation of mechanical allodynia and thermal hyperalgesia	[138,139]
	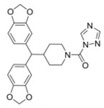 JJKK-048	<0.4 nM	Not tested	[123]
Dual MAGL FAAH	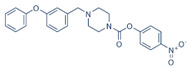 JZL195	13 nM FAAH 19 nM MAGL	Reduction of peripheral and cephalic pain	[145]
	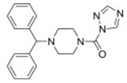 AKU-005	0.2–1.1 nM MAGL 63 nM FAAH	Not tested	[141]

Inhibitor potencies defined by IC_50_ values in rat brain membranes (OL135, URB597, URB937, JZL184, URB602, KML29, MJN110, JZL195), Colo cell line (PF3845) and rat cerebellar membranes (JJKK-048, AKU-005).

## 5. Conclusions

Migraine pain is a common and disabling condition which remains often intractable, and despite the huge number of patients debilitated by migraine pain, an effective therapy free of side effects is still lacking. Several recent studies suggest endoCBs as a new promising treatment for migraine pain given the overlap between ECS and key regions for the nociceptive system at most of the stages of pain signal generation, transmission, and perception. Therapeutically optimal levels of endoCBs AEA and 2-AG, aiming to provide analgesia but minimize the unwanted cannabimimetic effects, can be achieved by administration of emerging potent MAGL and/or FAAH inhibitors. The strength of this therapy relies on the specificity and selectivity of the compounds, confining their anti-nociceptive effects to sites where endoCB could be efficiently mobilized proportionally to the local neuro-immune activity. This field of research needs further investigation, which now become possible by combining various modern methods including highly sensitive ABPP assays to evaluate activities and the sensitivity to inhibition of endoCBs hydrolases, LC/MS spectrometry to determine endoCB levels in specific tissues, along with electrophysiological tools and behavioral testing in animals. Identification of novel treatments acting specifically on druggable molecular targets in the brain and in the peripheral meningeal trigeminovascular nociceptive system suggests a promising approach to control migraine pain, ultimately limiting the undesired side effects of new treatments.

## Data Availability

Not applicable.

## Abbreviations

2-AG	2-arachidonoyl glycerol
ACC	Anterior cingulate cortex
AEA	*N*-arachidonoyl ethanolamide, anandamide
BoNT-A	Botulinum neurotoxin serotype A
CB1/2	Cannabinoid receptors 1, 2
CGRP	Calcitonin gene related peptide
CNS	Central nervous system
CSD	Cortical spreading depression
DAGL	Diacylglycerol lipase
ECS	Endocannabinoid system
EndoCBs	Endocannabinoids
FAAH	Fatty acid amide hydrolase
MAGL	Monoacylglycerol lipase
mGluR	Metabotropic glutamate receptor
NAPE-PLD	NAPE-specific phospholipase D
NMDAR	N-methyl-D-aspartate receptors
PNS	Peripheral nervous system
TG	Trigeminal ganglion
TGVS	Trigeminovascular system
TRPM3	Transient Receptor Potential Cation Channel Subfamily M Member 3
TRPV1	Transient receptor potential vanilloid 1
VGCC	Voltage-gated calcium channel
